# Observations on Enlarged Tonsils

**Published:** 1848-01

**Authors:** Frank H. Hamilton

**Affiliations:** Buffalo


*Observations on Enlarged Tonsils.* BY FRANK H. HAMILTON, M. D., of Buffalo.—I have brief notes of 52 cases of enlarged tonsils which I have extirpated. The notes have been made chiefly by my students, and are not as full or systematic as I wish they were; yet the experience which they embody is perhaps worth recording. I have made some operations in addition to these in my note book, but in the following summary and conclusions, I shall endeavor to confine myself to such practical inferences as the *recorded* cases alone will justify.

*Pathology.*—In all the above cases the glands have been simply enlarged and slightly indurated, except that in six or eight instances a few small tubercular deposites have been found in them. I have never seen them scirrhous, or affected with any other malignant disease. Of some 50 or more preserved in alcohol, and in the College Museum, not the slightest difference can be seen in their structure; and as to size, the largest is not more than sixteen lines in length, and eight in breadth.

*Etiology.*—Among the causes assigned by the parents and friends, or by the patients themselves, 10 are set down as attributed to scarlatina, 7 to whoopiug cough, 3 to croup, 18 to hereditary predisposition, as shown in its having occurred in the parents or other members of the family, and the balance are unaccounted for. Many of these patients had a scrofulous look, and some had, at the same time, enlargements of the lymphatic glands of the neck.

*Age at which the Enlargement was first noticed.*—Generally between the third or fourth and seventh year of life : from which time they gradually increased in size until the tenth or fifteenth year, or until they were removed.

*Effects and results when left to themselves.*—They gradually diminish in size after the tenth or twelfth year, and finally disappear, or rather, become reduced to their normal size : so that it is extremely rare to see enlarged tonsils after the twentieth year. I have never seen but one after the twenty-seventh year, and this was at the forty-second year, but the enlargement was moderate

In the mean time, however, or, at least, during all the period of childhood, the patient is liable to frequent attacks of acute tonsilitis, which alone are sometimes sufficient to permanently impair the health; to a serious impediment in speech and hearing, both of which I think may become permanent, but in proof of this supposition I cannot cite any cases; it is certain, however, that it often interferes materially with the education of the child. Such children are almost always dull in their studies, or timid or petulant in their manners and feelings. They are also liable to severe attacks of croup in early life, and later to more chronic bronchial affections.

*Local or general Therapeutic treatment.*—Of this I have but little right to speak, since I have myself seldom resorted to any other means than extirpation ; and I have not, because I have seldom heard of a cure clearly traceable to these measures, but especially because I have found the operation so simple, certain and safe. If anything can be said in defence of therapeutic means, I would rather leave it to those who have themselves experienced their advantages.

*Circumstances contra-indicating an operation.*—The operation of excision ought not to be made when the glands are *inflamed*, unless the patient is threatened with suffocation, since the operation is then more difficult, more painful, and is more liable to be followed by fatal inflammation, (I have seen the operation made upon a child, whose tonsils were at the time inflamed, terminate fatally in a few days from inflammation extending to the larynx,) but especially because the danger from hemorrhage is then much greater. A friend of mine, a clergyman, applied to me to excise his tonsils, but I declined because they were inflamed. He went next day to New York city and called immediately upon a famous tonsil cutter, who shaved them off at once, and the wounds bled during three days. The bleeding was finally arrested by the hot iron, but not until life was almost extinct.

They ought no to be excised when no other reason can be assigned than that they are enlarged.

We should prefer not to make the operation, where the patient has a hemorrhagic diathesis.

*Circumstances which indicate an operation.*—It ought to be made when from their size, the patient is in danger of immediate suffocation, in whatever condition they may be. It ought to be made, having first removed all inflammation, when they produce deafness or impair speech, or occasion frequent attacks of tonsilitis, or occasional attacks of laryngitis, or endanger the developement of chronic bronchitis, or of phthsis in persons already predisposed. Or it might be proper where, as in one case, which will be stated hereafter, the glands were affected with an obstinate neuralgic disease.

*Age at which the operation can be made, and at which it is usually made.*—Three of my operations were made upon children two years old, but at this age owing to the smallness of the mouth, the operation is more difficult. I have made one upon a man aged forty-two, but the large majority have been made between the ages of four and ten years.

*Mode of operation.*—No one now questions the superiority of excision to the old, tedious and terribly painful process of ligation. The last argument upon which the ligature was sustained, viz.—that the knife was the most dangerous because of the hemorrhage which might follow—has long been given up: for we know that if some little danger does actually exist from the hemorrhage, it is much more than counterbalanced by the danger which attends the inflammation inevitably consequent upon the use of the ligature. I have never used the ligature myself, but I remember to have seen it used when I was an apprentice, and I can assure those who have never witnessed it, that it is one of the most barbarous operations in the surgical catalogue.

The question is now, only as to the *instrument to be used* in excision. The number of instruments invented for this purpose is very great, the majority of which have some real merit, and will answer, for probably every man will first use that instrument to which he is most accustomed, yet if I were to recommend a *young* practitioner to choose, I would unhesitatingly give preference to "Owen's" instrument. This is the instrument which I have always used, and have preferred, notwithstanding I have five or six other well made and ingenious instruments constructed for the same purpose. In it seem to be combined all the excellences, with none of the faults, of other tonsil instruments. Let me mention a few of the qualities which the instrument ought to possess.

The handle should be sufficiently large to be felt in the grasp and not too smooth—it should be set firmly on the shaft and at a proper angle, greater than a right angle. The shaft should be seven inches long, and three-fourths of an inch wide, so as to separate the teeth of the patient and protect the fingers of the operator—the tonsil should be seized by forceps attached to the instrument rather than by a pin, since, when the pin is used the tonsil may slip off after the operation, and be swallowed, or fall upon the rima glottidis and produce suffocation; the forceps should be so attached upon a pivot as that the tonsil can be drawn through the ring as much or as little as the operator chooses. Those instruments whose stilets merely transfix the tonsil without drawing it through, often fail of taking a sufficient amount of the gland. The forceps which are moved by the action of a spring, I have seen tear out—it is much better that the hand of the operator alone should control the forceps, and especially because by the hand alone can discretion be exercised to the amount to be removed. The teeth of the forceps, when the forceps are opened, should never encroach upon the inner circle of the ring. The ring or fenestrum which is to receive the tonsil ought to be of moderate size ; if large, it requires too much breadth at this part of the instrument. The size which will be found adapted to nearly all, if not all tonsils, is ten lines in breadth by twelve in length. Into this we can always introduce the gland sufficiently far to seize it with the forceps, and if seized, we shall never fail to be able to draw it through as much as we choose. The edge of the knife or guillotine must be camerated—roof-shaped and not rounded, and it must cut by "propulsion," being propelled by the thumb of the hand which holds the instrument. Instruments that cut by "retraction" cannot have properly shaped guillotines, nor can such shaped guillotines be easily sharpened, and they are objectionable also from the fact that they require one hand to hold the instrument, while the other retracts the blade, and the forceps must be abandoned. Besides this, it will always be found where one hand holds the instrument and the other withdraws the blade, that the two forces acting in opposite directions will not be equal, and the ring is liable to be pulled forward or pushed backward and to slip from the gland ; but when the thumb of the same hand which grasps the handle projects the blade, the antagonist powers are equal, and the instrument remains steady and firm to its place.

These are a few of the principal points in the construction of a tonsil instrument, whose value every one who operates much will appreciate, but there are many other little details which go to make the perfect instrument, and which require an experienced cutler to properly supply.

If the instrument is not perfect, I would rather use a long pair of forceps and a probe pointed bistoury, yet I object to these generally, because the operation with the bistoury is more difficult, and exposes the mouth and tongue to be badly cut, an accident which can never happen with a well constructed tonsil instrument.—(Owens' instrument may be had at his cutler's shop in Albany.)

The patient being seated before a strong light, the instrument is introduced with its "back" (by the "back" I mean that surface upon which the forceps lie, and by the "face," the opposite surface) applied to the tongue, and its "face" directed to the roof of the mouth : and in this way it is carried *below* the tonsil, and the tonsil is made to drop into it by pressing *from below*—a highly practical point, which constitutes nearly all the art of seizing the gland—the "face" is then turned obliquely upwards and outwards and pressed snugly upon the tonsil, while the forceps is made to seize it, and by steady traction draw it through. If the gland is large, the forceps should be moved laterally and slowly. The thumb now completes the operation by firmly thrusting the knife forward.

When, owing to the inability or disinclination of the patient to control the tongue, it is so thrust about that the tonsil cannot be kept in view, the forefinger of the hand not employed in holding the instrument may be held in the ring until the tonsil is *felt* to be fairly entered, and then the same hand may be withdrawn to seize the forceps, and the balance of the operation will be completed as before described. So easy is it to adjust the gland and seize it by the sense of feeling alone, that, as my students will remember, I seldom look into the mouth during the operation, and yet, if it is large enough to be seized at all, I never fail to bring it out, and so far as I am myself concerned, I have come to prefer this mode of operating, yet I cannot say that those unaccustomed to the operation would not operate better when the gland is in sight.

*Hemorrhage from the wound.*—The amount is generally trifling, usually not more than half an ounce—occasionally it is two or three onnces. If it does not cease spontaneously in a minute or two, a gargle of cold water will arrest it in most cases: but if this fail, let the neck be freely exposed, and a neck-cloth filled with snow placed about the neck, and especially opposite the seat of the tonsil. If snow cannot be obtained, pounded ice, or even cold wet cloths will answer. This plan I have never seen fail of arresting the bleeding most promptly, even where the most powerful astringents and caustics had already been resorted to with no effect, or with the effect only of increasing the hemorrhage at each application by disturbing the partially formed coagula. In only three cases of all the number operated upon, has the bleeding been alarming. The two first, a girl and a boy, both a little past the age of puberty, occurred, by a singular coincidence, on the same day. I had removed about two thirds of each gland in both patients. One bled until partial syncope occurred, and the other was finally arrested by the snow applied to the neck, and short of syncope. This was my first trial of the snow neck-cloth, and it succeeded after a full trial of a great variety of gargles, &c. The last case was a young lady aged 15, and of a strong hemorrhagic diathesis, of which I knew nothing until after the operation. Nor was I informed of the bleeding, which had only come on after she had reached home, until she was already much exhausted. The snow neckerchief again promptly arrested the bleeding, and it did not return.

From this accident therefore the operator has little to fear; nor need he apprehend more danger when he cuts off two-thirds, or even *the whole* of the gland, than when he merely shaves it or halves it. I have repeatedly, as many witnesses can testify, removed the gland *entire*, and not more than one or two tablespoons full of blood have followed; while the most alarming bleedings which have occurred were from wounds which left a portion of the glands *in situ*. No good reason then can be assigned why the gland should not be more fairly extirpated than has been usually recommended and practised.

*Other accidents.*—No other accidents are liable to follow a properly made operation. The inflammation is usually very slight, and seldom requires any treatment. In a week or ten days, the soreness is entirely gone.

*Results.*—If no good reason can be assigned why the gland should *not* be removed more fairly than is generally recommended, a sufficient reason can be assigned why *it ought* to be. When the whole or two-thirds of the gland is cut away, no more trouble is experienced from this source, but when one-half or one-third is removed, the balance does not generally disappear, and not unfrequently it again enlarges. This remark does not agree with the experience of some surgeons who have written upon this subject, and who, believing that if one-half or one-third is cut away the remainder will soon disappear, do not think it necessary to remove more than half at any time. But I have again and again seen cases in which the operation was thus imperfectly made, return to have them re-excised. Some of these have been my own cases, and in which I predicted at the time that the glands would be very likely to trouble them again. In no case, however, in which two-thirds has been removed, have the patients returned.

Neither the speech or the hearing are improved until after the lapse of months after the operation is made. I have never known the speech to be *injured* by the operation; the apprehensions which some have felt upon this score do not seem to me to be well founded.

